# Four drug metabolism-related subgroups of pancreatic adenocarcinoma in prognosis, immune infiltration, and gene mutation

**DOI:** 10.1515/med-2022-0433

**Published:** 2022-03-04

**Authors:** Tongyi Zhang, Liyong Zhu, Jianhua Cai, Jiaqi He

**Affiliations:** Department of General Surgery, Huadong Hospital Affiliated to Fudan University, Jing’an District, 200040, Shanghai, China; Department of General Surgery, Huadong Hospital Affiliated to Fudan University, No. 221 Yan’an West Road, Jing’an District, 200040, Shanghai, China

**Keywords:** pancreatic adenocarcinoma, drug metabolism genes, prognosis, immune infiltration, unsupervised clustering analysis

## Abstract

We aimed to screen the drug metabolism-related subgroups of pancreatic adenocarcinoma (PAAD) and to study the prognosis, clinical features, immune infiltration, and gene mutation differences of different subtypes in PAAD patients. All 181 cases of PAAD samples and clinical characteristics data were downloaded from The Cancer Genome Atlas (TCGA). After matching the drug metabolism-related genes downloaded from PMID 33202946 with the TCGA dataset, the drug metabolism-related genes were initially obtained. Besides, univariate Cox regression analysis was used to screen the drug metabolism genes related to the prognosis of PAAD. Moreover, the construction of the protein–protein interaction (PPI) network and gene ontology were performed. The four subgroups of PAAD obtained from unsupervised clustering analysis were systematically analyzed, including prognostic, GSVA, immune infiltration, and gene mutation analysis. A total of 83 drug metabolism genes related to the prognosis of PAAD were obtained and enriched in 16 pathways. The PPI network was composed of 248 relationship pairs. Four subgroups that can identify different subtypes of PPAD were obtained, and there were significant differences in survival and clinical characteristics, mutation types, and immune infiltration abundance between subgroups. A total of 17 different pathways among the four subgroups involved in cell cycle, response to stimulants such as drugs, and transmembrane transport. In this study, the four subgroups related to the drug metabolism of PAAD were comprehensively analyzed, and the important role of drug metabolism-related genes in the immune infiltration and prognosis of PAAD were emphasized.

## 1 Introduction

The death of pancreatic ductal adenocarcinoma, also known as pancreatic adenocarcinoma (PAAD), is one of the highly lethal cancer types [1,2,3]. Because PAAD is very difficult to prevent or diagnose early in the curable stage and the prognosis is not ideal, the diagnosis and staging of PAAD is the key to the treatment of this disease [4]. For the treatment of patients with PAAD, conventional combined chemotherapy has made significant progress in the treatment of PAAD. However, the subtypes of the disease present broad resistance to therapy [5]. Therefore, comprehensive and accurate diagnosis and treatment strategies such as personalized and targeted therapy based on tumor and genomic markers have great application prospects [6].

The in-depth understanding and research of PAAD-related gene regulation can provide a theoretical basis for the molecular targeted therapy of PAAD. KRAS proto-oncogene, GTPase (KRAS), and tumor protein p53 (TP53) were confirmed to be important biomarkers for the prognosis of PAAD, and can also be used as a tool for treatment prediction [7]. It was well known that TP53 was a tumor suppressor, and mutations of TP53 can be detected in 70% of PAAD patients [8]. The mutation of TP53 in PAAD patients mainly led to the loss of DNA binding ability, which in turn resulted in the loss of gene transcription activation [9]. For example, compared with patients whose TP53 function was completely lost due to mutations, patients with normal TP53 expression had significantly improved survival [10]. Therefore, it is suggested that TP53 can be used as a biomarker for the prognosis of PAAD and treatment prediction. As for KRAS, it is a small GTPase (21 kDa) and 95% of PAAD patients had KRAS mutations [11], which caused KRAS to be constitutively activated resulting in uncontrolled cell proliferation and other processes that led to the development and spread of cancer [12]. The results of multiple studies have shown that compared with patients with wild-type KRAS, patients with KRAS mutations showed worse responses to gemcitabine or erlotinib and worse survival [13,14]. These molecular markers may play an important role in the future treatment of PAAD. However, the research and application of PAAD drug metabolism genes in the field of therapy and prognosis are still very limited.

Compared with molecular targeted therapy, immunotherapy has little effect on PAAD, not only because of its immunosuppressive tumor microenvironment but also because of the unclear role of immune cells in PAAD [15]. The screening of immune cells related to the clinical characteristics of PAAD may have guiding significance for the early diagnosis of PAAD patients. Studies have shown that the expression of CD8^+^ T cells was correlated with the survival time of PAAD [16]. In particular, high tumor infiltration of CD8^+^ T cells can lead to a better prognosis. In addition, the synergistic activation of T and natural killer (NK) cells in a transgenic mouse model of resectable PDAC has been shown to prevent the recurrence of PAAD [17]. Therefore, detecting the expression of immune cells may be important for judging the prognosis of patients with PAAD.

**Figure A1:**
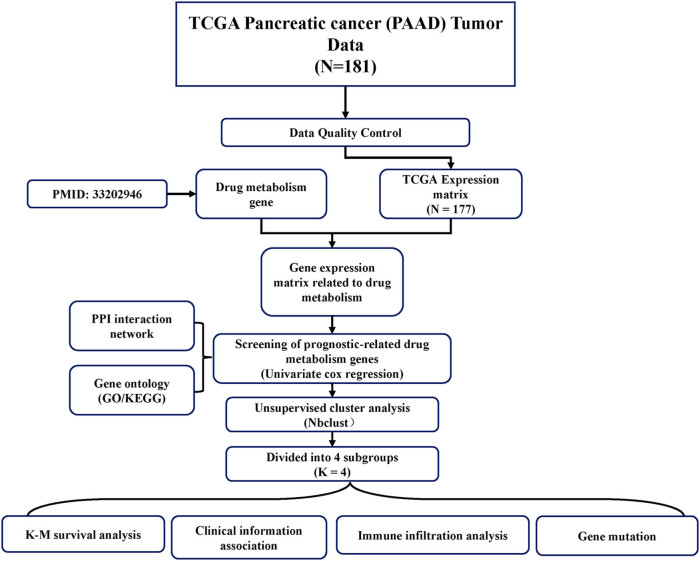
Flow chart.

Currently, there is no joint study of drug metabolism-related genes with the immune infiltration and prognosis of PAAD. In this research, PAAD tumor data were downloaded from The Cancer Genome Atlas (TCGA), and multiple data mining methods were used to further screen drug metabolism-related genes and analyze PAAD subgroups that were related to the prognosis of PAAD (Figure A1). This research may contribute to exploring the relevance between the drug metabolism-related genes and the prognosis of PAAD as well as immune infiltration of PAAD.

## 2 Materials and methods

### 2.1 Data acquisition and screening

A total of 181 PAAD case samples and clinical data were downloaded from the TCGA dataset (http://xena.ucsc.edu) [18], and 177 PAAD samples included in this study (all tumors samples) were obtained after excluding the adjacent samples (TCGA.H6.A45N.11A, TCGA.H6.8124.11A, TCGA.YB.A89D.11A, TCGA.HV.A5A3.11A). For the data quality control (QC), the count value of the PAAD gene expression matrix was used for log2(count + 1) standardization, and then 60,489 transcripts were merged with the same transcripts through Python (the mean method for processing). After removing the genes whose expression level was 0, 31,186 Ensembl_IDs were obtained. Gencode (ftp://ftp.sanger.ac.uk/pub/gencode/Gencode_human/) was used for gene annotation of the transcriptome. In addition, the conversion between Ensembl_IDs and Symbol ID was performed on Python. The “normalizeBetweenArrays” function in the R language limma 3.9.19 package was used to normalize the data [19]. Finally, 298 drug metabolism-related genes were downloaded from PMID 33202946. Besides, through matching with the TCGA data set (PAAD) and eliminating genes with missing values less than 50%, the drug metabolism-related gene expression matrix was set for subsequent clustering studies.

## 3 Screening of prognostic related genes

In order to screen the drug metabolism genes related to the prognosis of PAAD, the R language Survival Package (version3.10.3, http://www.bioconductor.org/packages/release/bioc/html/survival.html) was used for univariate Cox regression analysis based on the sample survival information of the TCGA data set and the gene expression value in each sample [20].

### 3.1 Construction of protein–protein-interaction (PPI) network and pathway enrichment analysis

In order to explore the interaction of the expressed proteins of drug metabolism-related genes, PPI prediction analysis was performed on the prognostic-related drug metabolism proteins obtained above. STRING (v9.1) (https://www.string-db.org) was applied in the construction of the PPI network, and a threshold (score threshold = 0.4) was set to draw the online protein interaction network. In addition, the PPI score (score threshold = 0.4) was adopted to show the PPI interaction relationship more clearly. The Cytoscape (v3.8.2) tool software (https://github.com/cytoscape/cytoscape.js/tree/v3.8) was used to draw the network diagram.

The R language ClusterProfiler (v3.16.1) package (http://bioconductor.org/packages/release/bioc/html/clusterProfiler.html) was used for pathway enrichment analysis [21]. Moreover, through gene ontology (GO) and Kyoto Encyclopedia of Genes and Genomes (KEGG) databases, pathway enrichment analysis was performed on prognostic-related drug metabolism genes, and the false discovery rate (FDR) method was used to correct the *P* value. Finally, the ClusterProfiler (v3.16.1) package was used to draw the pathway enrichment map.

### 3.2 Unsupervised clustering analysis

To further determine the regulation mechanism of different drug metabolism-related genes in patients with PAAD, unsupervised clustering analysis was performed by the R language NbClust package (v 3.0), which was used in all TCGA samples (177 cases) (https://cran.r-project.org/web/packages/NbClust/index.html) to determine the best classification group [22]. Then, clustering was performed on the k-mean method provided by the Nbclust package. Finally, principal components analysis (PCA, R language dplyr package v 1.0.5, https://cran.r-project.org/web/packages/dplyr/, prcomp function) and linkage clustering analysis (R language pheatmap package v 1.0 .12, https://cran.r-project.org/web/packages/pheatmap/) were used to explore the differences between different subgroups.

### 3.3 Gene set variation analysis (GSVA) of different subgroups

In order to explore the differences in the pathway scoring status of the four different subgroups, we used GO (http://software.broadinstitute.org/gsea/msigdb) as a reference set and conducted the subgroup of 177 samples through the ‘gsva’ method provided by the R language GSVA (v 3.10.3, http://www.bioconductor.org/packages/release/bioc/html/GSVA.html). First, linkage cluster analysis was used to explore whether different subgroups have associated clustering patterns on GSVA scores and whether there was a linkage between subgroups. If linkage existed, subgroups were considered to be combined and participated in the analysis of the pathway score. Limma package (v 3.44.3) of R was used to analyze the different pathways among subgroups.

### 3.4 Prognostic survival analysis and clinical information association analysis among subgroups

The R language Survival (v3.2-10) package (Version3.10.3, http://www.bioconductor.org/packages/release/bioc/html/survival.html) was used for prognostic risk difference analysis based on the survival matrix of the subgroups and subtype groupings. In addition, the Kaplan–Meier (K–M) survival prognostic curve (*P* value < 0.05) was used to explore the difference between different subgroups.

Then, TCGA clinical data were used to count the number of WHO grades (G), sex ratios, age distributions, and responses to chemotherapy (complete response, CR; partial response, PR; progressive response, PD; stable disease, SD) included in each subtype. In addition, the Chi-square test in the MASS (v 7.3-53.1) package of R language was used to calculate whether there were differences in different clinical characteristics of each subgroup. Because each subgroup contained different number of people, the above clinical characteristics were counted as “percentage = count/total number of people” as the final index included in the study. Finally, ggplot2 was applied in the drawing of bar graphs.

### 3.5 Analysis of the abundance of immune infiltration in subgroups

In order to explore the differences in the abundance of immune infiltration between subgroups, 177 TCGA samples were included in this part of the study. Immune infiltration analysis was performed by the CIBERSORT (R 4.0.2) package [23], which used a leukocyte signature matrix (LM22). Besides, a variety of immune cell types in all PAAD samples were analyzed by the deconvolution method [24]. After calculating the proportion of immune infiltrating cells, the Mann–Whitney U-test was used to compare and analyze the differences in the proportion of immune infiltration of patients in different subgroups.

Furthermore, we searched for markers of different M2 macrophages subtypes (M2a, M2b, M2c, M2d) from the published literature [25,26]. The number of markers for M2a, M2b, M2c, and M2d are 6 (CD163, CD200R1, CD206, TGM2, IL1R2, CD209), 2 (CD163, CD86), 3 (CD163, TLR1, TLR8), and 2 (VEGF, IL10), respectively. Next, based on the expression matrix of all genes, the ssGSEA (single sample enrichment analysis) algorithm was used to estimate the relative infiltration level of each M2 macrophages subtype. Then, the *t*-test was used to compare whether there was a significant difference between the M2 macrophages subtypes of each two clusters. *P* < 0.05 was considered as a statistically significant threshold.

### 3.6 Analysis of gene mutations in subgroups

In order to explore the differences in gene mutations between different subgroups, PAAD genome Maf files (somatic mutations) and corresponding clinical signature files in the TCGA database were adopted. At first, the genomic Maf files of different subgroups were separated. Then, the maftools package of R language was used to perform gene mutation analysis [27], and the mutation waterfall chart of different subtypes was drawn. Finally, the mutation information of different subgroups was counted. Furthermore, we selected the top 2 genes with mutation frequency in each subgroup and analyzed the KEGG pathways of these genes that were significantly different between mutant and nonmutant samples.

## 4 Results

### 4.1 Eighty-three drug metabolism genes were related to the prognosis of PAAD

A total of 177 cases of TCGA expression matrix were included in this study (Table S1). The TCGA expression matrix included 31,186 genes and the clinical phenotypes, namely, WHO grade, gender, age, response to chemotherapy (SD, PD, CR, and PR), TNM classification (TNM), and tumor stage ((Table S2).

After the intersection of the 298 reported drug metabolism genes with TCGA (Table S3), a total of 270 genes were used as the drug metabolism-related gene expression matrix for subsequent clustering studies (Table S4). The results of univariate Cox regression analysis between the survival data of 177 samples in TCGA and 270 drug metabolism genes suggested that 83 genes had a significant correlation with the prognosis of PAAD (*P* < 0.05) (Table S5).

### 4.2 PPI network construction and pathway enrichment analysis results

**Figure 1:**
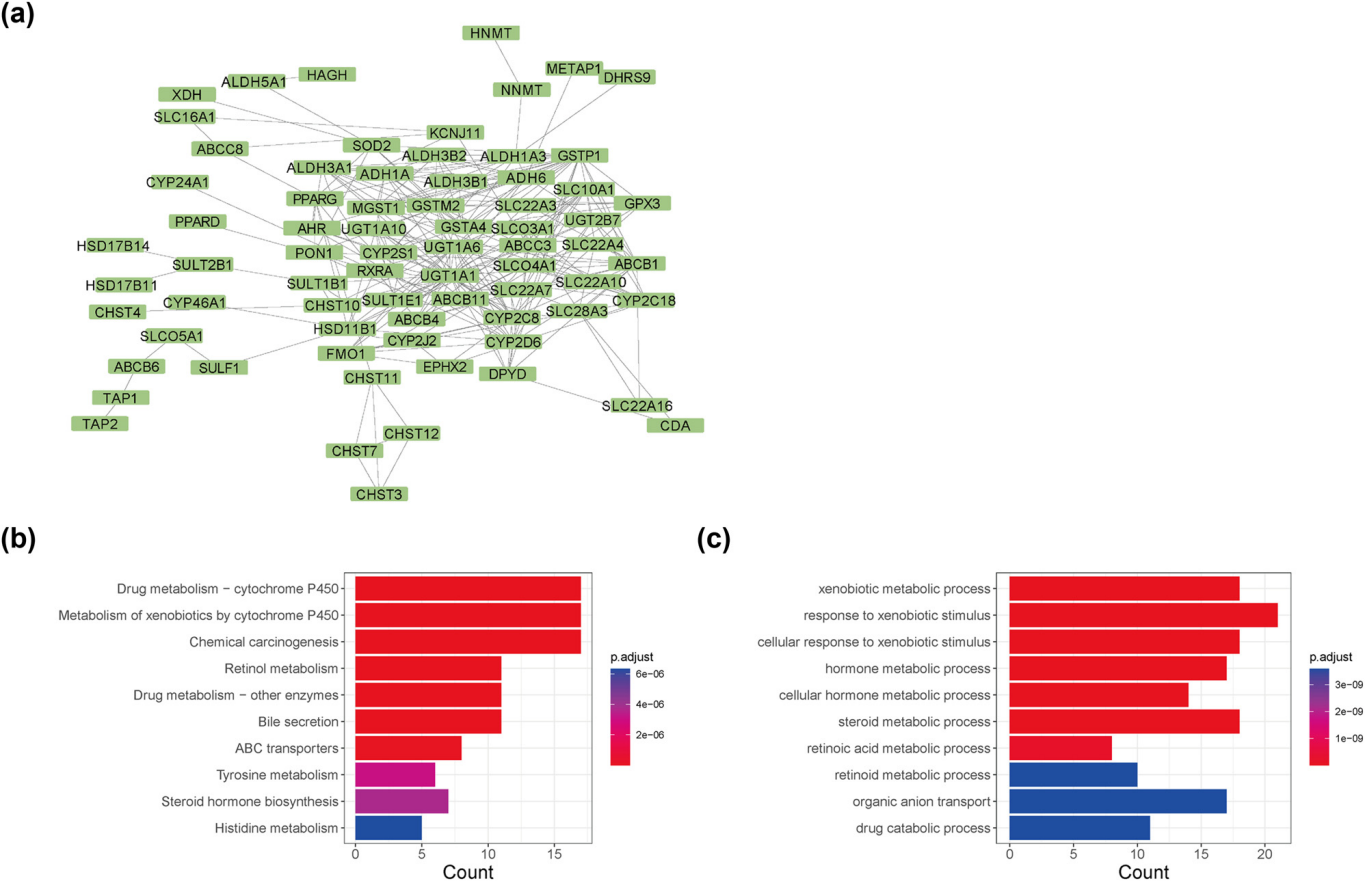
Protein–protein interaction network (PPI) and enrichment analysis of 83 drug metabolism genes: (a) PPI network; (b) enrichment results of Kyoto encyclopedia of genes and genomes (KEGG); and (c) enrichment results of gene ontology (GO).

The PPI network of 83 PAAD prognosis-related drug metabolism proteins was constructed using Cytoscape (Figure 1a). A total of 248 interactions relationship pairs were obtained with an interaction score >0.4 (Table S6). It can be seen that in the PPI network the two relationship pairs with the highest degree of connection were ABCC8 (ATP binding cassette subfamily Cc member 8) – KCNJ11 (potassium inwardly rectifying channel subfamily j member 11) and PPARG (peroxisome proliferator activated receptor gamma) – RXRA (retinoid × receptor alpha).

Besides, the enrichment analysis of KEGG and GO pathways was performed on the 83 genes, and a total of eight KEGG and eight GO pathways were enriched. The eight enriched KEGG pathways were as follows: drug metabolism-cytochrome P450, metabolism of xenobiotic by cytochrome P450, chemical carcinogenesis, retinol metabolism, drug metabolism-other enzymes, bile secretion, ABC transporters, and tyrosine metabolism (Figure 1b). Moreover, the GO pathway included xenobiotic metabolic process, response to xenobiotic stimulus, cellular response to xenobiotic stimulus, hormone metabolism, cellular hormone metabolic process, steroid metabolic process, retinoic acid metabolic process, and drug catabolic process (Figure 1c).

### 4.3 PAAD subgroups were obtained from unsupervised clustering analysis

**Figure 2:**
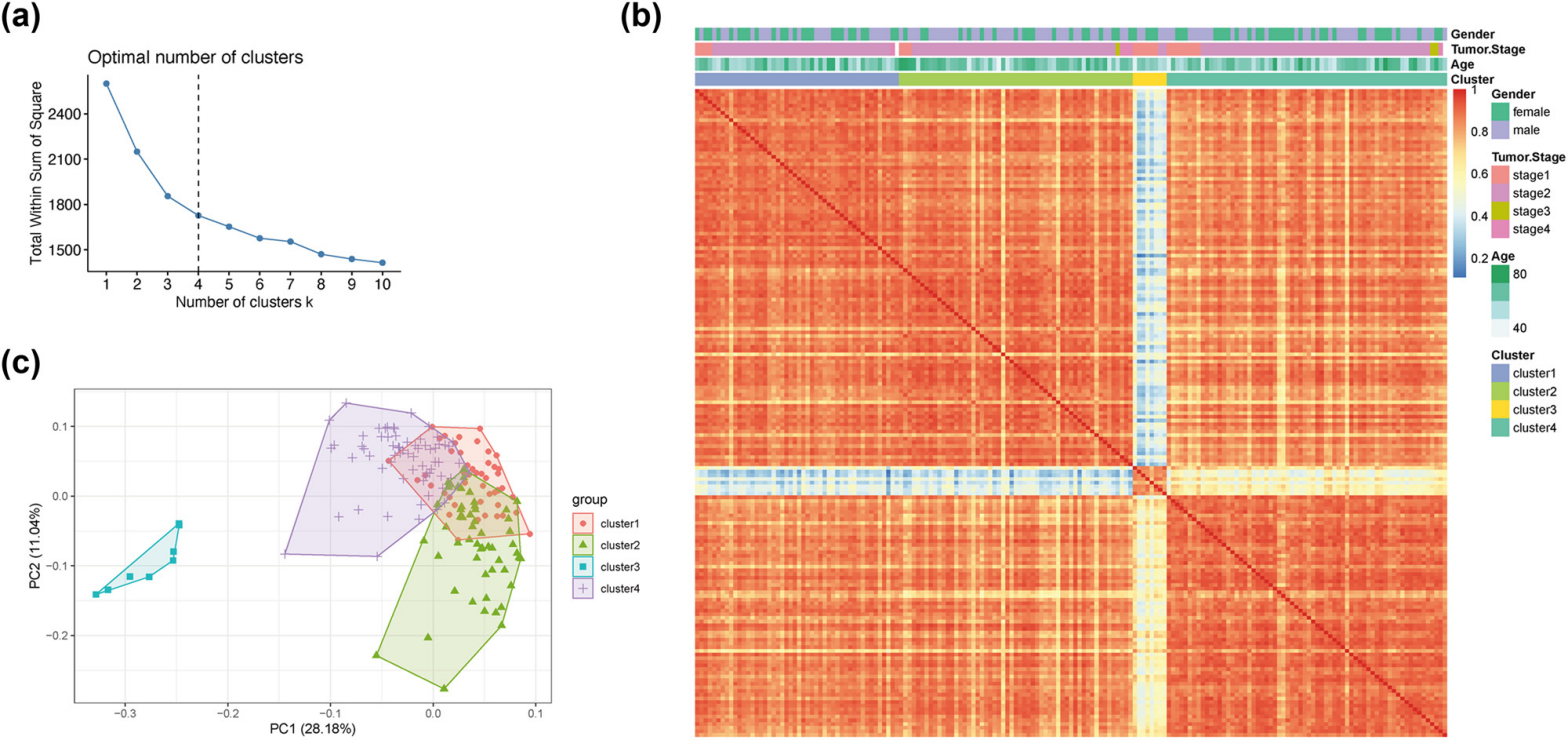
Unsupervised clustering of 83 drug metabolism genes: (a) the best type was determined; (b) linkage association clustering analysis (Clusters1–4); and (c) principal components analysis (PCA) of subgroup clustering.

Based on the 83 drug metabolism-related genes, patients were divided into different subgroups through unsupervised cluster analysis. As shown in Figure 2a, as the number of clusters increased, the total within sum of square (WSS) gets smaller. When the WSS decreased slowly, the effect of further increasing the number of clusters was considered not to be better, and this point was the optimal number of clusters. The final result showed that the samples can be divided into four subgroups (Figure 2a). Moreover, Cluster 1, Cluster 2, Cluster 3, and Cluster 4 contained 48, 55, 8 and 66 people, respectively (Table S7), and the linkage clustering heat map revealed that although there was a certain correlation between samples in different groups, the correlation between the same subgroup was greater, and the correlation was also reflected in different clinical characteristics (Figure 2b). Furthermore, it can be seen from PCA that the four groups were separated well (Figure 2c, Dim1 = 28.1%).

### 4.4 Prognostic K–M survival analysis and clinical information association analysis results

**Figure 3:**
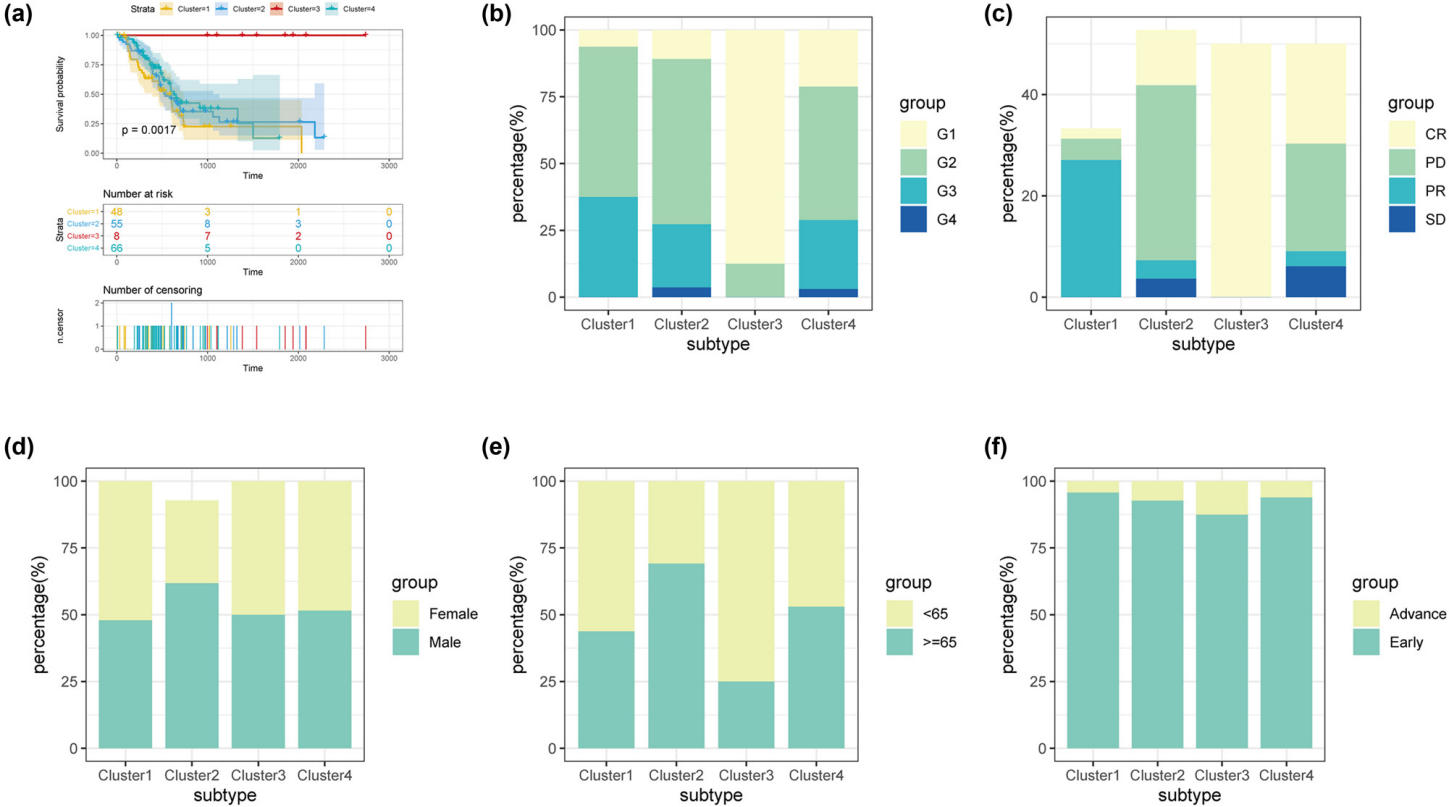
Prognostic analysis and clinical phenotype of the subgroup: (a) Kaplan–Meier survival analysis; (b) the distribution of WHO classifications in different subgroups; (c–f) distribution of chemotherapy response (c), gender (d), age (e), and tumor stage (f) in the four subgroups (because 53% of the samples in Figure 4c lacked data on chemotherapy response, each group of samples cannot constitute 100% ratio).

For the above four subgroups (Clusters 1–4), the survival data of TCGA were used for K–M survival analysis. The results suggested that there were significant differences among the four subgroups (Figure 3a, *P* = 0.0017), confirming that the obtained subgroups were real. In addition, the results of clinical information association analysis showed significant differences in the four subgroups (Figure 3b–f). These differences were reflected in the WHO classification (G1, G2, and G3), gender ratio, survival status, and response to chemotherapy (CR, PR, PD, and SD) (Table 1, *p* < 0.001). In the Cluster 1 subgroup, there were more individuals with the age of <65 years, WHO G2 grade, and PR, but the survival rate was relatively lower. Similar to Cluster 1, in Cluster 2, the WHO classification results showed that G2 > G3 > G1 and the survival rate was relatively low. The difference was that more individuals in this subgroup were >65 years old and PD. Moreover, the age of all patients in the clusters was less than 65. All patients in Cluster 3 alive were alive with CR. After WHO classification, the G1 level had a clear advantage. In Cluster 4, the proportion of SD was slightly higher than that in other subgroups.

**Table 1:** Comparison of clinical characteristics distribution differences among different subgroups of TCGA

Phenotype		Cluster 1	Cluster 2	Cluster 3	Cluster 4	*P* value
Age	<65	56.25	30.91	75.00	46.97	0.00^***^
	≥65	43.75	69.09	25.00	53.03	
State	Alive	39.58	38.18	100.00	56.06	0.00^***^
		60.42	61.82	0	43.94	
Tumor stage	Early	95.83	92.73	87.50	93.94	0.34
	Advance	4.17	7.27	12.5	6.06	
Gender	Female	52.08	30.91	50.00	48.48	0.07
	Male	47.92	61.82	50.00	51.52	
WHO	G1	6.25	10.91	87.50	21.21	0.00^***^
	G2	56.25	61.82	12.50	50.00	
	G3	37.50	23.64	0.00	25.76	
	G4	0.00	3.64	0.00	3.03	
Chemotherapy response	CR	2.08	10.91	50.00	19.70	0.00^***^
	PR	27.08	3.64	0.00	3.03	
	PD	4.17	34.55	0.00	21.21	
	SD	0.00	3.64	0.00	6.06	

***P value <0.001.

### 4.5 GSVA analysis of different subgroups

**Figure 4:**
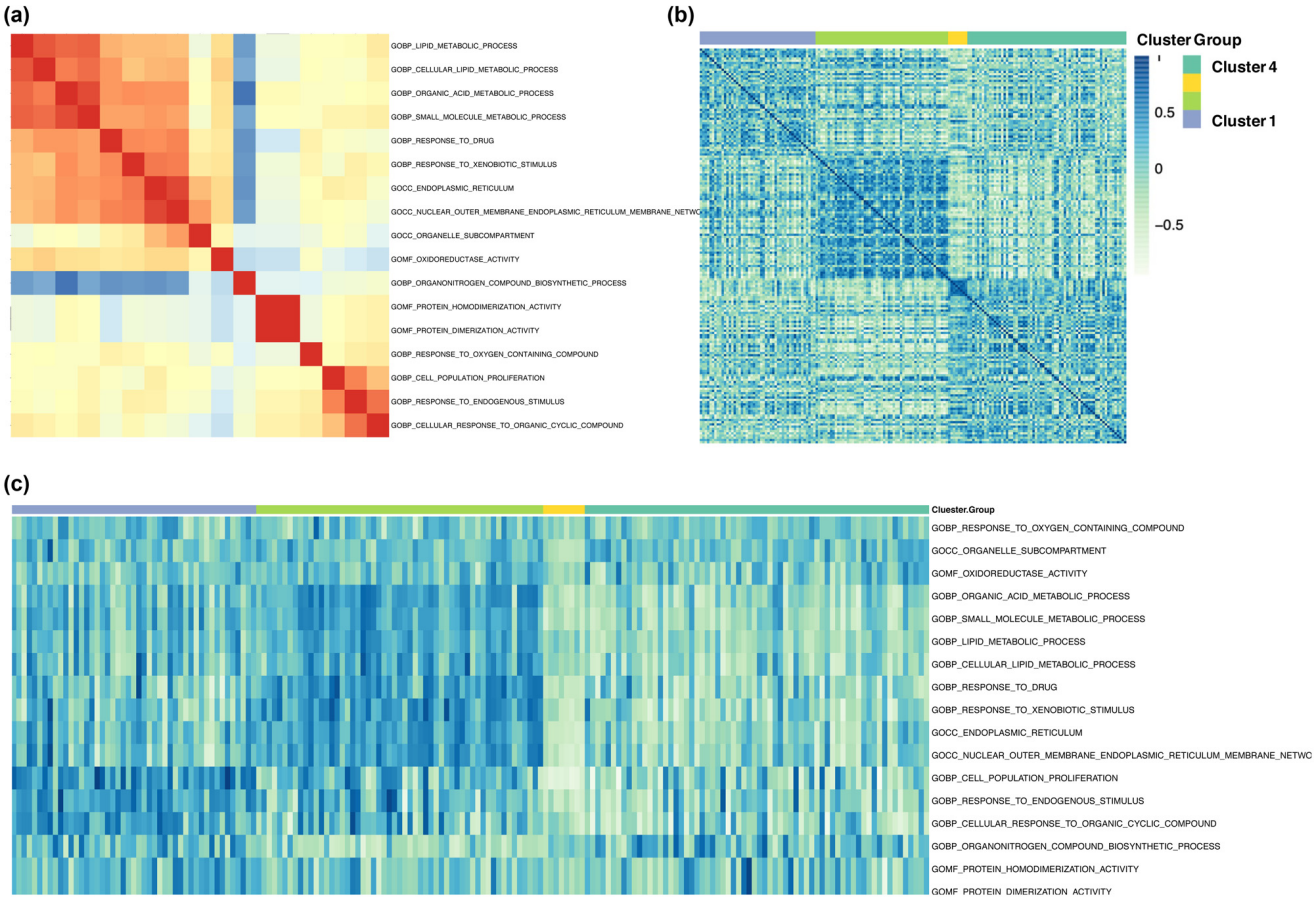
Gene set variation analysis: (a) the linkage-heatmap describes the existence of linkage correlation modules between the GSVA scores of the GO pathway; (b) the linkage-heatmap describes the correlation modules that are linked between the GSVA scores within different subtypes of TCGA; (c) significant differences in GO pathways (*P* value <0.05) in the heatmap can see obvious stratification in the four subgroups.

The correlation between the significantly enriched GO pathways was explored through linkage association cluster analysis (Figure 4a). From the perspective of subgroups, we can clearly see that the four subgroups formed interlocking modules, indicating significant differences in GO pathways between the four groups (Figure 4b). In addition, multigroup square difference analysis was used to explore significantly different GO pathways among the four subgroups, and the results indicated 17 significantly different pathways including cell cycle, metabolism and synthesis of organic matter, response to stimulants such as drugs, and transmembrane transport (Figure 4c).

### 4.6 Abundance analysis for immune infiltration among subgroups

**Figure 5:**
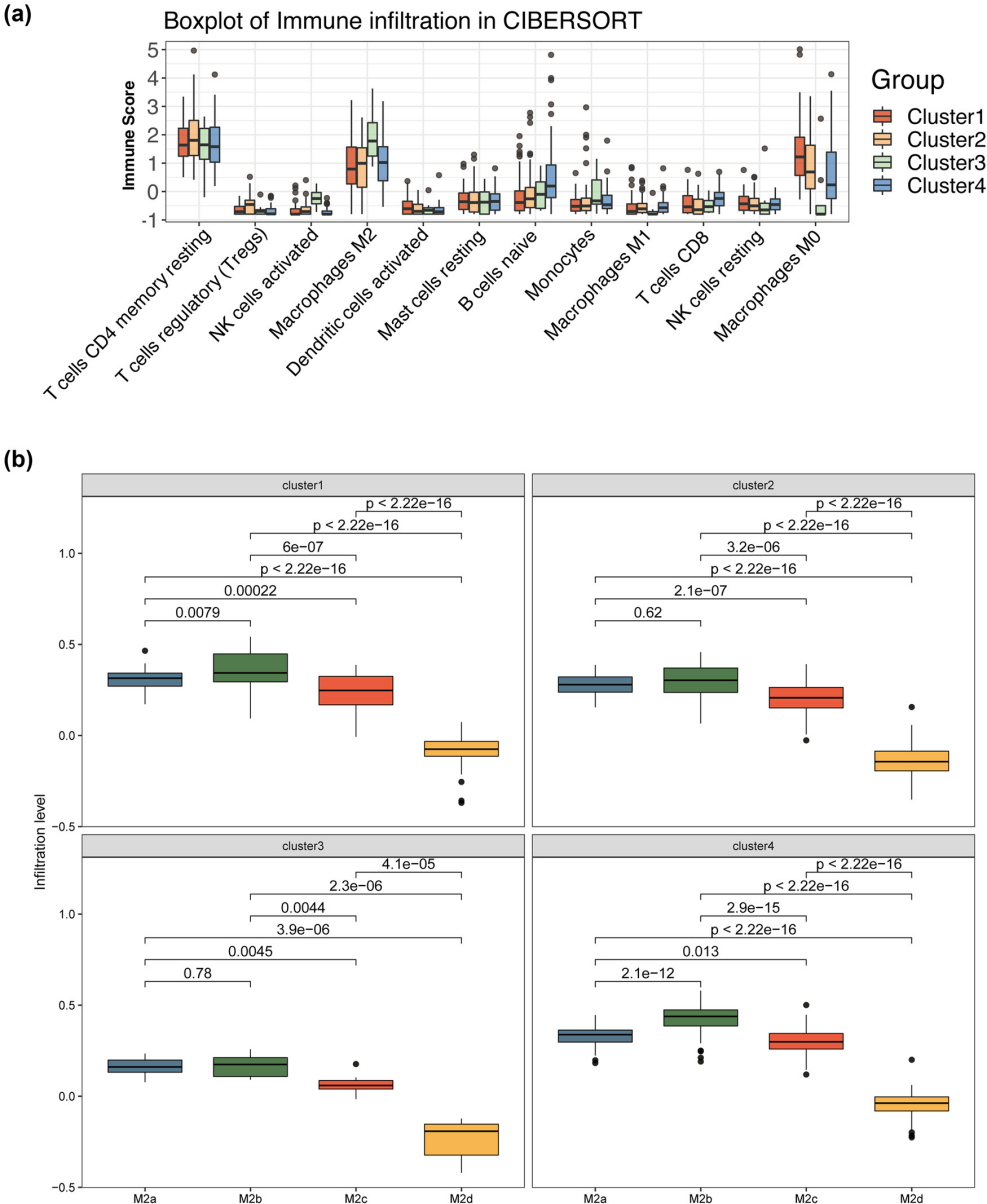
Analysis of the differences in immune infiltration among subgroups: (a) immune scores of 12 kinds of immune cells in different subgroups; and (b) infiltration levels of M2a, M2b, M2c, and M2d in subgroups.

The CIBERSORT algorithm was used to calculate the 22 immune infiltration abundances among the above subgroups, and more than 50% of immune cells with no immune abundance were excluded. Finally, 12 immune cells were included in the study (Table S8). Furthermore, the 12 immune cells were subjected to the Whitney U-test (Table S9). The results indicated that the immune score of immune cells varies among different subgroups (Figure 5a). Compared with the other three clusters, M2 macrophages had the highest immune score in Cluster 3, while M0 macrophages and M1 macrophages had the lowest immune score in Cluster 3 (Figure 5a). Further analysis of different subtypes of M2 macrophages (M2a, M2b, M2c, M2d) showed that the infiltration levels of the four subtypes were basically consistent among different subgroups (Figure 5b). In the same subgroup, M2b macrophages had the highest infiltration level, followed by M2a macrophages, then M2c macrophages, and finally, M2d macrophages.

### 4.7 Gene mutation analysis for subgroups

**Figure 6:**
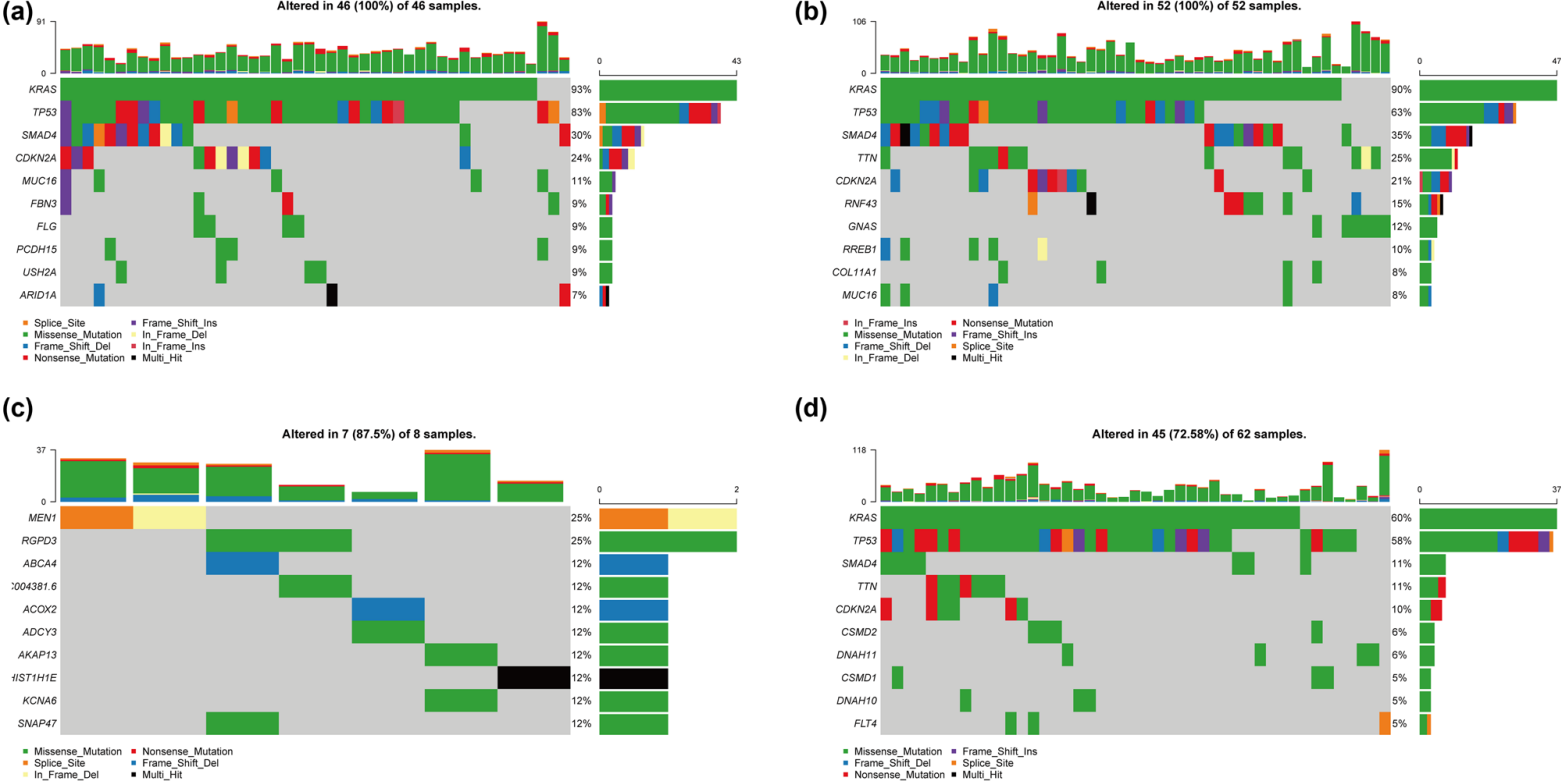
Somatic mutation analysis of Cluster 1 (a), Cluster 2 (b), Cluster 3 (c), and Cluster 4 (d).

Four sets of different mutation waterfall charts were drawn to count the mutation information of different subgroups (Figure 6, Table 2). The results showed that there were differences in the mutant genes and the mutation frequency among the 4 subgroups (Table 3). Besides, KRAS and TP53 with the highest mutation frequency in Clusters 1, 2, and 4 did not appear in the Top 10 mutation frequencies of the Cluster 3 subgroup.

**Table 2:** Statistics of the number of mutant genes in different subgroups

Type	Cluster 1	Cluster 2	Cluster 3	Cluster 4
Missense mutation	224	302	12	77
Nonsense mutation	9	11	1	9
Total	234	313	13	86

**Table 3:** Top 10 mutated genes in different subgroups

	Cluster 1	Frq	Cluster 2	Frq	Cluster 3	Frq	Cluster 4	Frq
Top1	KRAS	0.19	KRAS	0.53	CYP2C8	0.13	TP53	0.08
Top2	TP53	0.08	TP53	0.11	ESRRB	0.13	KRAS	0.06
Top3	FLG	0.06	GNAS	0.07	FN1	0.13	CDKN2A	0.03
Top4	C2orf16	0.06	ADAMTS15	0.04	GANAB	0.13	AGXT	0.02
Top5	APOB	0.04	ADAMTSL3	0.04	MACF1	0.13	AHNAK	0.02
Top6	FCRL5	0.04	NOS3	0.04	NSUN7	0.13	AKAP6	0.02
Top7	KCNH7	0.04	PSD	0.04	PRDM15	0.13	AOC1	0.02
Top8	NEB	0.04	TENM2	0.04	SNAP47	0.13	APBA2	0.02
Top9	PLEKHH2	0.04	TGFBR2	0.04	SRSF6	0.13	AQP4	0.02
Top10	SPTA1	0.04	TMEM108	0.04	TRIM47	0.13	ATP9A	0.02

Frq = Mutation frequency.

**Figure 7:**
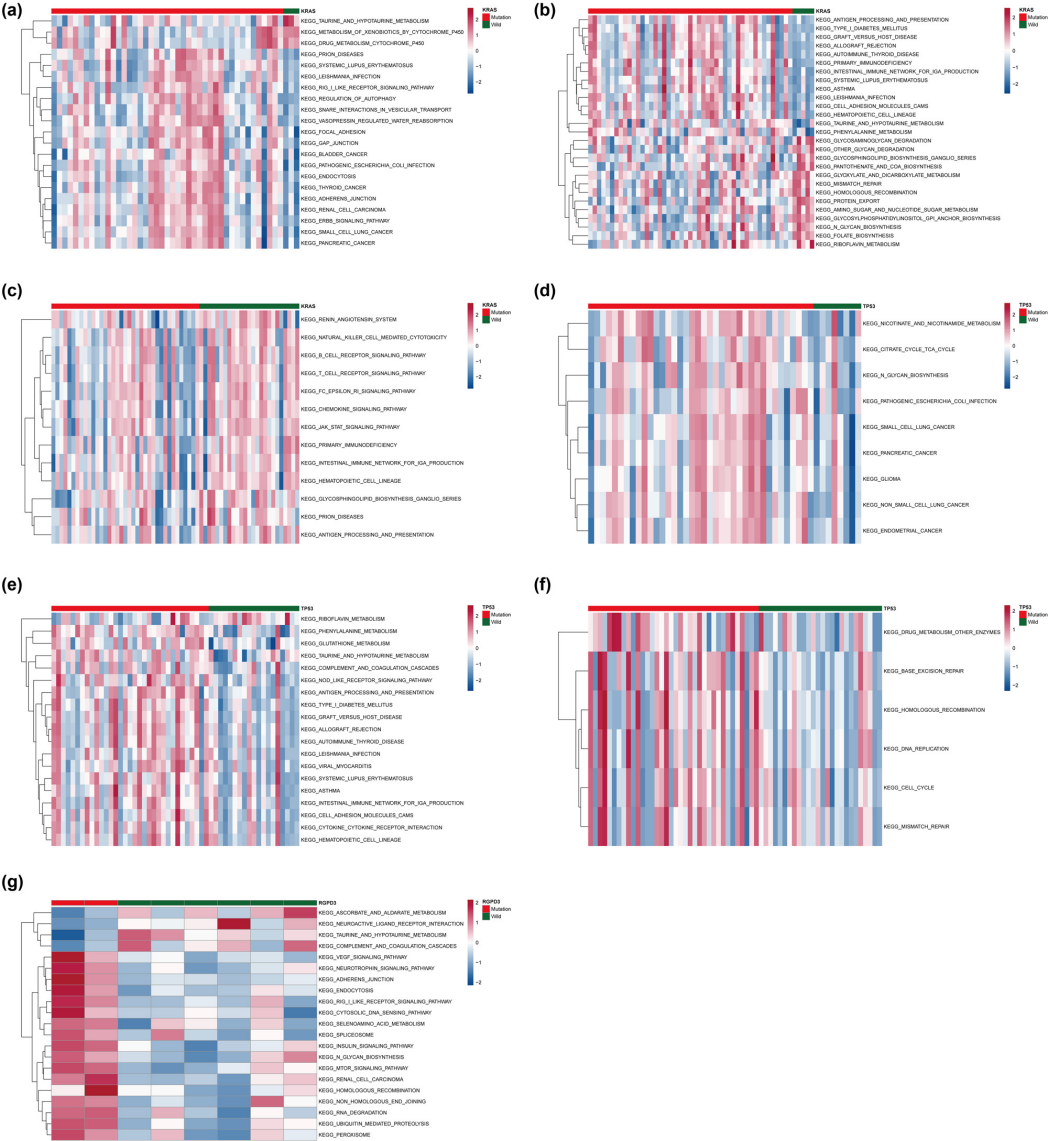
Significant differences in KEGG pathways between samples with genetic mutations and nonmutated samples. Significant differences in KEGG pathways between KRAS-mutated and -nonmutated samples in Clusters 1 (a), 2 (b), and 3 (c). Significant differences in KEGG pathways between TP53-mutated and -nonmutated samples in Clusters 1 (d), 2 (e), and 3 (f). Significant differences in KEGG pathways between RGPD3 mutated and nonmutated samples in Clusters 3 (g).

To further analyze whether the genes with missense mutation might have functional changes and further affect the pathway, we selected the genes with high missense mutation frequency (KRAS, TP53, RGPD3) in different subgroups for further analysis. The pathways with differences between mutant and nonmutant KRAS samples in Cluster 1 included taurine and hypotaurine metabolism, regulation of autophagy, pancreatic cancer, etc. (Figure 7a). KRAS mutations in Cluster 2 involved changes in pathways such as taurine and hypotaurine metabolism, immunodeficiency, antigen processing and presentation (Figure 7b). For Cluster 4, there were 27 patients with mutations in the KRAS (Figure 6). There were differences in multiple pathways between patients in the KRAS gene mutation group and patients in the nonmutation group, including immunodeficiency, natural killer cell-mediated cytotoxicity, JAK stat signaling pathway, etc. (Figure 7c). TP53 mutations in Cluster 1 patients may involve changes in pathways including pancreatic cancer and TCA cycle (Figure 7d). In Cluster 2, patients with TP53 mutation and nonmutation had changes in multiple pathways, including phenylalanine metabolism, antigen processing and presentation, etc. (Figure 7e). For TP53 mutations in Cluster 4, there were some changed pathways between mutant and nonmutant patients, including base excision repair, homologous recombination, cell cycle, etc. (Figure 7f). We only detected RGPD3 mutations in Cluster 3 patients. According to whether RGPD3 was mutated or not, the patients in Cluster 3 were divided into the mutation group and nonmutation group. It was found that there were differences between various pathways, including ubiquitin-mediated proteolysis, taurine and hypotaurine metabolism, MTOR signaling pathway, etc. (Figure 7g).

## 5 Discussion

In recent years, although there have been advances in treating PAAD, no joint research of drug metabolism-related genes with the immune infiltration and prognosis of PAAD has been performed. In this study, we screened prognostic differentially expressed genes related to drug metabolism of PAAD using data from public databases. We also constructed a PPI network and made an enrichment analysis for these genes screened above. Furthermore, unsupervised cluster analysis was used to divide these genes into four subgroups, and there were differences in survival, clinical, immune infiltration, and gene mutation among these subgroups.

In the PPI network constructed by 83 PAAD prognosis-related drug metabolism proteins, the relationship pair with the highest connection degree in the PPI network diagram was ABCC8-KCNJ11. However, the effect of their interaction on the prognosis of PAAD has not been reported before this study. ABCC8 was a member of the ATP-binding cassette (ABC) transporters family. It is well known that the higher expression levels of a large number of ABC transporter genes were associated with an increased chance of survival in patients with PAAD. However, ABCC8 was only confirmed to be an independent prognostic factor of glioma and has not been detected in any studied cell lines of PAAD [28]. Regarding KCNJ11, it is a member of the potassium channel gene family and interacted with ABCC8 to form ATP-sensitive potassium (KATP) channels mediating the secretion of insulin [29]. Besides, studies have shown that decreased co-expression of ABCC8-KCNJ11 may increase the risk of diabetes [30,31]. It is speculated that interacting proteins ABCC8-KCNJ11 may be involved in the prognosis of PAAD and can be used as a new prognostic factor for PAAD. In the PPI network, PPARG – RXRA showed the second-highest degree of connectivity. PPARG is a ligand-activated transcription factor that formed a heterodimer with RXRA. Research data show that both PPARG and RXRA were related to the characteristics of PAAD [32]. Moreover, it was found that PPARG (not RXRA) was an independent prognostic indicator.

After the unsupervised clustering analysis, four subgroups were obtained and Cluster 3 had a significantly better prognosis compared with the other three subgroups (*p* < 0.05). It was speculated that the good prognosis of Cluster 3 was not only because Cluster 3 had the most patients in the G1 stage and all of them showed a CR but also had a younger age. The abundance of immune infiltration between groups also showed differences. The abundance of M2 macrophages and activated NK cells in Cluster 3 were the highest and the abundance of M0 macrophages was the lowest in the four groups. It is well known that different phenotypes of macrophages have been demonstrated to play distinct roles in tumor progression [33]. M2 macrophages were divided into different subtypes by different stimuli, including M2a, M2b, M2c, and M2d. M2a activation is obtained by stimulating macrophages with IL-4 or IL-13 [34]. M2b cells were elicited by stimulation with LPS or IL1beta. Unlike M2a cells, M2b cells produce large amounts of TNF-α, IL-1β, and IL-6 in addition to IL-10. M2c cells were elicited by IL-10, GC, or TGF-β, and play an important role in the early stages of wound healing [35]. M2d cells were induced by A2AR signaling pursuant to initial stimulation by TLR agonists [36]. In this study, we compared the infiltration level of different M2 macrophages in the same subgroup and found that the infiltration level of M2b was the highest in the four subgroups. A previous study has found that an increase in the proportion of specific tumor-associated macrophages characterized by M2b can lead to acquired resistance to bevacizumab [37]. However, the role of the high infiltration level of M2b in PAAD patients needs further research in the future.

Furthermore, the mutation detection results of subgroups 1, 2, and 4 all showed a higher proportion of KRAS and TP53 mutations but these two mutations in cluster 3 did not appear in top10. The presence of KRAS mutation is generally associated with clinical aggressiveness of cancer and reduced survival of the patient [38]. A study has reported that KRAS and TP53 prognosis of PAAD is directly associated with a specific mutation of KRAS [39]. These results also further confirmed that the PAAD subgroup clustering and association analysis based on drug metabolism-related genes were effective. To further understand the role of gene mutations in different subtypes, we selected the genes with high missense mutation frequency (KRAS, TP53, RGPD3) in different subgroups. The results indicated that mutations in these genes may involve different pathway changes between different subgroups. Taurine and hypotaurine metabolism, regulation of autophagy, phenylalanine metabolism, and other pathways have been confirmed to be involved in the occurrence of PAAD [40,41,42]. In addition, in this study, the two genes KRAS and TP53, which have a higher mutation frequency in a variety of cancers, did not appear in Cluster 3 patients, which is an interesting point. First of all, the classification of patient subgroups in this study was based on gene expression, and clinical characteristics were not included in the analysis. Therefore, the result is an unbiased analysis. Second, our cluster analysis included genes related to drug metabolism and genes that complete response to chemotherapy may be more similar in expression patterns, which leads to clustering such patients into a subgroup (Cluster 3). Patients in Cluster 3 will eventually have a better prognosis because of their good response to drugs.

The GSVA analysis of the differences between the four subgroups showed that the pathways with significant differences were mainly involved in the cell cycle, metabolism and synthesis of organic matter, response to stimulants such as drugs, and transmembrane transport. Mammalian cell cycle regulation is a very complicated process. The loss of function of key regulatory points can lead to uncontrolled cell proliferation and further tumors [43]. The difference in response to drug stimulation between different subgroups is consistent with our screening of drug metabolism-related goals, and in the analysis between subgroups, we also found that different groups had different responses to chemotherapy. The next research plan is to expand the number of samples and enrich the types of samples (including PAAD samples of different nationalities, etc.) for further confirming the role of data mining analysis and clustering analysis of drug metabolism-related genes on the prognosis and immune infiltration of PAAD disease. In addition, the highly connected drug metabolism-related gene relationship pairs in the PPI network need to be further verified in different PAAD subgroups.

## 6 Conclusion

In this study, through TCGA PAAD tumor data mining analysis, we constructed the PPI network composed of PAAD drug metabolism-related proteins and four different PAAD subgroups to investigate the possible molecular pathways related to the prognosis and immune infiltration of PAAD. The relationship pairs ABCC8 – KCNJ11and PPARG – RXRA may be associated with the good performance on the PAAD prognosis. The genes involved in these relationship pairs may provide a basis for in-depth research on the prognosis mechanism of PAAD.
